# Empowering Chinese college students in English as a foreign language writing classes: Translanguaging with translation methods

**DOI:** 10.3389/fpsyg.2023.1118261

**Published:** 2023-04-17

**Authors:** Limei Zhang

**Affiliations:** Singapore Centre for Chinese Language, Nanyang Technological University, Singapore, Singapore

**Keywords:** Translation method, EFL writing, writing performance, confidence and interest in writing, Chinese college student

## Abstract

Learning activities based on translation has attracted increasing attention among researchers in English as a Foreign Language (EFL) teaching and learning under the influence of translanguaing pedagogies. This study was designed to examine the effect of translation methods as pedagogical tools on students’ writing performance in EFL classrooms. 89 Chinese college students participated in the study. They were required to complete tests of essay writing before and after the use of the translation method. Nine students were invited to attend an interview after the writing test. Results showed that the students improved their essay writing performance significantly after the translation method. The participating students’ confidence and interest in essay writing were also enhanced. Findings from the study have important implications for effective writing instruction for Chinese EFL college students.

## 1. Introduction

Learning activities on the basis of translation is gaining increasing attention among researchers in the area of English as a Foreign Language (EFL) teaching and learning under the influence of translanguaging pedagogies (Atkinson, 1987; Liao, 2006; Calis and Dikilitaş, 2012; Jiménez et al., 2015; Lee, 2018; García and Kleifgen, 2019). Translangauging, according to García (2009), is the “multiple discursive practices in which bilinguals engage in order to make sense of their bilingual worlds” (García, 2009, p. 45). Translangauging pedagogies, as a result, encourage readers and writers to tackle and create texts with their diverse language resources to deepen understanding and improve language proficiency (García, 2009; García and Kleifgen, 2019; Wei and Lin, 2019).

Researchers have thus conducted studies which adopt translation as a English as a Second Language (ESL) or EFL learning strategies. House (2014) argued that translation is a process through which “a text from one language is reproduced in another language” (p. 1). Different from traditionally held viewpoint, the cognitive process of translation, which involves the comparison of two languages, is recently perceived to be able to help EFL students structure English grammar and vocabulary systematically and alleviate their anxiety in learning new languages (Lee, 2018; García and Kleifgen, 2019). For example, Liao’s (2006) study showed that translation plays a positive and facilitative role in EFL college students’ learning experience. Lee (2018) argued that translation methods improved the writing performance of students who had low English proficiency level.

Although translation proves to be useful in EFL teaching and learning, few studies have been conducted to examine the effect of translation methods as pedagogical tools in EFL classrooms (Calis and Dikilitaş, 2012). To turn translation into an effective and systematic tool used in EFL teaching and learning, more empirical studies should be carried out to provide supportive evidence for the actual practice of translation methods in EFL learning activities (Lee, 2018). The current study was therefore designed to fill the gap in the area of translation methods used in EFL classrooms, especially the effect of translation methods in EFL writing instruction.

Writing well is of critical importance for success in academic performance and professions. For college students, effective writing ability is essential for them to communicate with teachers, peers and future colleagues and co-workers. One having good writing ability tends to have good overall language proficiency. On the other hand, writing also promotes students’ ability to articulate ideas and synthesize perspectives. For example, good writing ability can help enhance language learners’ overall language proficiency as good writing must conform to the conventions of grammar and usage of the target language. However, attaining writing proficiency is often difficult. As such, it is necessary and imperative for language educators to find more effective methods to improve students’ writing ability.

With China’s policy of further opening-up and extensive exchange and communication with western countries in commerce, culture, science and technology, there is an increasing demand for competent English users in a variety of professions (Zhang et al., 2013; Zhang et al., 2014). Considering the fact that the ability to write effectively is of critical importance for students’ success in many situations and that acquiring English writing ability is often difficulty, researchers and practitioners have been exploring effective methods to enhance Chinese EFL college students’ writing ability (e.g., Wang and Wen, 2002; Zhang and Huang, 2020).

This study therefore attempts to investigate the effect of translation methods on Chinese EFL college students’ writing performance in linguistic and affective dimensions, especially under the circumstances that pedagogical translanguaging is increasingly gaining attention recently. Findings from the study will have important implications for effective writing instruction for Chinese college students. With the translation method of writing instruction, it is likely to gain insights into the possible use of L2 learners’ overall language resources to improve their writing proficiency as well as their interests and confidence in writing English. More importantly, this study will also provide useful information for the use of translation-based writing instruction in EFL classrooms and give specific strategies in using pedagogical translanguaging as a strategy in learning and teaching L2.

## 2. Literature review

This section discusses relevant theories and empirical research findings in the areas of models of writing process, language transfer in second language learning and, studies on translation methods in EFL, and ESL teaching and learning.

### 2.1. The model of writing process

Researchers have conducted many studies on the writing process (Hayes and Flower, 1980; Wang and Wen, 2002). The consensual view is that writing is a complex activity consisting of various sub-processes in cyclical and varying patterns. In terms of the first language (L1) writing, Hayes and Flower (1980) proposed an influential model of the writing process. In this model, cognitive processes were subdivided into planning, transcribing, and reviewing. The cognitive process of planning was divided into setting goals, generating ideas, and organizing ideas into a plan. Revising was broken into evaluating and revising text. However, the three operational processes of planning, transcribing and reviewing have often been criticized for giving a false impression that writing is linear and flows step by step as it seems that there are clear boundaries among the three cognitive processes in writing (Wang and Wen, 2002).

Based on Hayes and Flower (1980) and their research findings, Wang and Wen (2002) proposed a model of L2 writing. Different from Hayes and Flower (1980), the mental activities during the composing process include task-examining, idea-generating, idea-organizing, text-generating and process-controlling. Before and/or during drafting, writers start the task-examining activities to read and analyze the writing requirements to ensure they have a good understanding of the task requirement. The idea-generating and idea-organizing activities are involved when writers plan their writing which is followed by text-generating activities upon the completion of the planning stage. In addition, writers monitor the whole process of writing constantly. As a L2 writing model based on empirical data, Wang and Wen’s writing model provides the theoretical framework for the current study.

### 2.2. Translation and L2 teaching and learning

A consensual view among researchers is that second language (L2)^1^ learners use their first language (L1) strategically while learning the L2 (Cummins, 1979; Ellis, 1994, 2003; Kuo and Anderson, 2010). According to the influential Developmental Interdependence Hypothesis (Cummins, 1979), language and literacy skills developed in learners’ first language (L1) can be positively transferred during the process of L2 learning (Kuo and Anderson, 2010). Ellis (1994, 2003) proposed a framework of the role of L1 and L2 communication and learning. In his framework, language transfer is a very important component. According to this framework, when L2 learners are engaged in comprehension and production activities using their L2, they make use of their L1 system and the transfer from L1 to L2 takes place. In addition, it was found that transfer of learning was influenced by a series of cognitive and linguistic factors such as the distance between L1 and L2, learners’ language proficiency and educational settings etc. (Chung et al., 2018). In L2 writing, language transfer can be a learning device as well as a strategy to resolve problems in writing (Wang and Wen, 2002; Cenoz and Gorter, 2021).

On the basis of theory of language transfer in L2 learning, researchers argued that L2 learners’ L1 should be attached more importance in L2 teaching and learning (Atkinson, 1987; Wang and Wen, 2002; Kim, 2011; Pan and Pan, 2012; Kellerher, 2013). For example, Atkinson (1987) suggested that as a potential useful resource, L2 learners’ mother tongue should play a more important role in learners’ fluency development, especially through learning activities based on translation. Auerbach (1993) argued that the practice of using English only in L2 instruction is “neither conclusive nor pedagogically sound” (p. 9). With a growing body of evidence, she pointed out that the use of L1 in L2 teaching and learning is effective and beneficial for L2 learners. Further, Malmkjaer (1998) argued that the use of translation in language teaching should be practised in a way closer to the actual use of translation in daily life to maximize the benefits of translation methods in L2 acquisition. Paradis (2007) argued that it might be faster for L2 learners to learn the translation equivalent for a concept in L1 than to learn a new label. In a word, translation is more and more perceived as a useful and beneficial method of teaching and learning in L2 classrooms.

### 2.3. Studies about translation methods in L2 teaching and learning

Researchers have also conducted empirical studies to investigate the effect of translation methods on L2 learning. For example, Carreres (2006) examined second and third year college students’ perception about using translation as a language learning tool using a 11-item questionnaire. She found that translation exercises were perceived as effective language learning activities in L2 teaching and learning by the participating students. Liao (2006) investigated 351 Taiwanese EFL college students’ learning beliefs about translation and their methods of using translation to learn English using questionnaire surveys and qualitative interviews. Results showed that translation plays a positive role in most students’ English learning processes, especially for the less proficient learners. Translation is perceived as a useful learning strategy in many students’ English learning. Calis and Dikilitaş (2012) reported on a classroom-based study on 28 Turkish elementary learners’ views on the use of translation as L2 learning practice using questionnaire surveys and interview questions. They found that the participants had a positive perspective on the use of translation as a learning and teaching tool in classroom practice. In other words, translation can be used as a means of promoting L2 learners’ language skills.

Similarly, Huang (2003) investigated the effect of cross translation on Taiwanese college students’ awareness of idiomatic English learning. Students were required to translate the Chinese of Chinese/English bilingual storybooks into English and then compare their own version of English with that of the book. Through a systematic comparison and analysis, students’ “Chinese English” was improved gradually. The results indicated that cross translation improved students’ awareness of English idiomatic expressions in that it could supplement the teaching of idiomatic expressions in classrooms. In addition, the cross-translation method also enhanced students’ awareness of unidiomatic English, improved their word usage and enriched their cross-linguistic knowledge of English usage.

In another study concerning EFL learners, Kim (2011) examined the use of grammar translation methods in a reflective and collaborative mode in an EFL writing classroom and its effect on students’ writing performance. Twenty Korean college students with relatively low English proficiency were invited to participate in the study. They were required to translate their own composition into Korean first and at the same time provided their reflective responses to the translating process. In the second phase, the participating students collaborated with their peers who translated their writing into Korean and then the participants did a second round of reflection. Results showed that students’ L1 proved to be a useful tool in L2 writing classrooms and the translation method of writing instruction helped improve the participants’ writing performance and made them view their own writing more positively and objectively.

More recently, Lee (2018) discussed the effect of the implementation of a translation method in EFL writing instruction on students’ writing performance and their attitudes toward writing. Thirteen Korean high-school students with low English proficiency participated in the study over three consecutive semesters. In line with the framework of action research, five stages of a translation writing method were designed for the study, including brainstorming, writing in Korean, writing in English and editing and rewriting. Results showed that the participating students’ writing performance was improved and at the same time they built up their confidence in English essay writing. This study suggests that translation can be used as an effective teaching method in EFL writing classroom, especially for low proficiency students.

As shown in the above-mentioned studies, translation has the potential to play an important role in L2 language teaching (Liao, 2006; Calis and Dikilitaş, 2012). However, few empirical studies have examined the positive aspects of translation methods in EFL contexts except Huang (2003), Kim (2011), and Lee (2018). To provide further insights into the effectiveness of translation methods, more empirical studies should be conducted to ensure that the method is more operationalized and easier to be deployed for teachers and students (Carreres, 2006). This study was therefore designed to investigate the effect of translation methods on Chinese EFL college students’ writing performance in linguistic and affective dimensions in terms of English writing.

The current study will address the following three overarching research questions:

1. Did the translation method improve Chinese college students’ writing performance in the linguistic dimension?
2. How did the translation method affect the writing performance of the students with higher and lower language proficiency?
3. Did the participants perceive the translation method improved their writing in the affective dimension?

## 3. Research methodology

### 3.1. Study design

This study adopted a quasi-experimental design. There were two groups of participating students: an experimental group and a control group. For the experimental group, the translation method of essay writing was used, while for the control group, there is no intervention. A test was conducted followed by an intervention of a translation method of essay writing. By the end of the intervention, both groups of students completed a second test of essay writing.

### 3.2. Participants

89 first-year Chinese college students from a university in the western part of China participated in the study. The western part of China is believed to be less developed than other parts of China, and the college students in western universities have a relatively low English language proficiency compared with those from the universities in other parts of China. For these students, English was a compulsory course in the first two years of their four year undergraduate programs. Before enrolling in their English courses at the beginning of their first school year, the freshmen were required to take a proficiency test to decide on their English proficiency level. A retired version of the College English Test Band 4 (CET4) was used as the test paper. As one of the most influential college tests in China (Jin, 2008), the CET is administered by the National College English Testing Committee on behalf of the Chinese Ministry of Education (Zheng and Cheng, 2008; Zhang et al., 2014; Zhang, 2018).

Due to limited resources, convenient sampling was used in choosing the participants, who were from two intact classes taught by the same English teacher. There were 14 female students (15.7%) and 75 male students (84.3%). They have received formal instruction in English as a foreign language for more than six years. Their ages range from around 18 to 23 years old with a mean age of 21 years old. Among them 47 were from the experimental group and 42 were from the control group. The language proficiency levels of the two groups were not significantly different from each other with *t* (87) = −1.87, *p* = 0.07. Based on the proficiency test, the students from the experimental and control groups were each divided into three groups of high-, medium- and low-proficiency for further analysis.

Among the participants, on the basis of their proficiency test, three high-, three medium- and three low-proficiency students were invited to attend an interview after the study. They were supposed to provide their feedback on the effect of the translation method on their writing. The information of the nine students were shown in Table 1.

**Table 1:** Information of the interview participants.

No	Gender	Language proficiency
1a	Female	High
1b	Male	High
1c	Male	High
2a	Male	Medium
2b	Male	Medium
2c	Male	Medium
3a	Male	Low
3b	Male	Low
3c	Female	Low

### 3.3. Instruments

There were two types of research instruments used in the study: writing tasks and interview questions.

#### 3.3.1. Writing tasks

The participating students were required to complete tests of essay writing before and after the intervention. The topics of the two essays were identical in terms of difficulty level and requirement of word limits following the curriculum. For the scoring of the essays, Jacohs et al.’s (1981) rubrics were adopted, which measure students’ writing in terms of content (30%), organization (20%), language (25%), vocabulary (20%), and mechanics (5%). The rating scale was chosen as it was regarded as “one of the best known and widely used analytic scale in ESL” (Weigle, 2002, p. 115) as its content and construct validity were well supported (Grabe and Kaplan, 1996).

#### 3.3.2. Interview

Nine students, of which three are of high English proficiency level, three medium level and three low level based on the language proficiency test, were invited to attend a 20-min interview after they completed the second test of essay writing. The purpose of the interview was to have a thorough understanding of the effect of the translation method on students’ writing performance. There are five questions in the interview which tap into students’ perspectives on the effect of the translation method on their English writing in linguistic and affective dimensions. The interview questions were shown in the Appendix.

### 3.4. Procedure

This section gives a description about the procedures of class instruction and the data collection.

#### 3.4.1. Class instruction procedure

All the participating students were required to complete a test of essay writing before they took part in the study. Then the experimental group started the lessons of essay writing class using the translation method. As shown in Table 2, there were five stages in the process of each writing class, including brainstorming, writing in Chinese, writing in English, editing and rewriting and submitting (Lee, 2018).

**Table 2:** The stages of the translation method in writing classes.

Stages	Time (min.)	What the students did	What the teacher did
1. Brainstorming	20	Discussed the given subject in L1 or L2; Jotted down key points in L1 or L2;	Made sure that students participated in the discussion actively; Helped students to remove barriers to the expression of ideas while they made the plan
2. Writing in Chinese	20	Wrote about the topic in Chinese Translated the Chinese draft into English	Showed the students that it was best to write clearly for easy translation
3. Writing in English	20 + 20	Step 1: Translated their Chinese draft into English Step 2: Completed the translation and submitted	For Step 1, prevented the students from using any dictionaries. For Step 2, allowed the students to use a dictionary. Helped the students with word choices and sentence structures, etc. in the process of translation. Provided feedback to the students on their translation.
4. Editing and rewriting	40	Undertook editing sessions Rewrote the essay using English	Helped students with their choices of words and expressions in English.
5. Submitting		Submitted the essays	Commented on and scored the students’ essays

Adapted from Lee (2018).

At the stage of brainstorming, students were required to discuss the given subject in their first language (L1) or second language (L2) and meanwhile they jotted down important ideas for their essay writing. At this stage, the teacher ensured that all the students participated in the discussion actively and provided help and guidance to the students individually or as a group whenever necessary. At the second stage, the students were required to write the designated essay in Chinese and then translate it into English. The teacher was supposed to provide guidance in terms of how to select appropriate and sufficient points and vocabulary in essay writing. From the third to fifth stage, students wrote the same essays in English before they edited and rewrote them for submission, while the teacher provided feedback on students’ translation and writing. The intervention, which was embedded in their regular English classes, lasted for around twelve weeks.

Different from the experimental group, there were roughly four stages in the process of writing for the control group, including brainstorming, writing in English, editing and rewriting and submitting.

#### 3.4.2. Data collection procedure

Before the data collection, ethical clearance was made and consent forms were signed by the participating students. Before the intervention started, the participants were required to complete a test of essay writing. At the end of the intervention, the participating students were invited to attend a second test of essay writing. Toward the end of the study, nine participants of three different English proficiency levels (i.e., high-, medium-, and low-level) were interviewed to provide their perspectives on the effect of the translation method on their writing performance. They were allowed to use either Chinese or English in the interview.

### 3.5. Data analysis

For the data analysis, descriptive statistics were calculated first. The author and a research assistant scored the essays. To ensure the consistency and reliability of the data analysis, the inter-rater reliability of the scoring, which is indicated using correlation index Pearson’s *r*, was calculated.

Next, a one-way analysis of covariance (ANCOVA) was conducted to examine the effect of the translation method on the participating students’ writing performance in terms of students’ overall writing score, content, organization, vocabulary, language use and mechanics. Scores of the first essay were the covariates. Partial eta square (*η*^2^) was estimated to evaluate the magnitude of effect sizes (small = .01; medium = .06; large = .14; Tabachnick and Fidell, 2019).

Further, effect sizes (i.e., Cohen’s *d*; small = .20; medium = .50; large = .80) were calculated to examine the differences of students with different language proficiency in terms of overall writing score, content, organization, vocabulary, language use, and mechanics (Cohen, 1988).

It should be noted that eta square represented the effect size for group differences, with the post-intervention writing scores as the dependent variable, whereas Cohen’s *d* represented the effect size for higher and lower proficiency groups’ gain scores from the first writing test to the second.

Nine interviewers’ (i.e., three high-, three medium- and three low-language-proficiency students) reports were first transcribed. For the analysis of interview data, we first constructed the coding scheme on the basis of the theoretical framework and the research questions and then re-checked the codes through iterative discussions. The interview data were then analyzed using Nvivo 12 computer program (QSR International, 2021) by the author and a research assistant. Cohen’s Kappa was generated by Nvivo on the basis of the two raters’ coding.

The purpose of the qualitative data analysis is to examine if the translation method improved students’ English writing in linguistic and affective dimensions of essay writing. In linguistic dimensions, the effect of the translation method on the content, organization, language use, vocabulary, mechanics of students’ writing product were examined. The effect on their writing process (i.e., task-examining, idea-generating, idea-organizing, text-generating and revising) was also investigated. For the affective dimension, the effect of the translation method on students’ interest and confidence in essay writing was examined.

## 4. Results

### 4.1. Descriptive statistics

Descriptive statistics was conducted which includes the means and standard deviations (SD) of the two essay-writing tests as well as their language proficiency, as shown in Table 3. The inter-rater reliability of the scoring, which is indicated using correlation index Pearson’s *r*, is .88. Cohen’s Kappa, which is the index of inter-rater agreement of qualitative data analysis, was.90, indicating that the data analysis was very consistent and reliable.

**Table 3:** Descriptive statistics of writing tests.

	Mean		SD	
	Exp	Con	Exp	Con
1st_content	19.53	19.26	2.14	1.57
1st_organization	15.40	15.87	2.15	1.32
1st_vocabulary	14.10	14.95	1.72	1.11
1st_language use	14.48	15.13	2.65	1.53
1st_mechanism	2.90	2.76	0.84	0.75
1st_overall_score	66.40	67.97	7.89	5.20
2nd_content	20.89	15.97	1.05	1.78
2nd_organization	16.47	15.12	1.00	0.99
2nd_vocabulary	16.21	14.88	0.95	1.02
2nd_language use	16.83	15.30	1.26	1.02
2nd_mechanism	3.53	3.52	0.50	0.57
2nd_overall_score	73.94	64.79	3.91	4.26
Lang_prof	70.65	73.66	6.77	8.33

Exp, the experimental group; Con, the control group; 1st, the first test; 2nd, the second test; Lang-prof, language proficiency.

### 4.2. ANCOVA

The analysis results of ANCOVA revealed that there were significant differences in students’ overall writing performance between the experimental and control group with *F* (1, 68) =92.52, *p* < .05, partial eta square (*η*^2^) =.58, and *F* (1, 68) =204.76, *p* < .05, partial eta square (*η*^2^) =.75 in content; F (1, 68) = 39.41, *p* < .05, partial eta square (*η*^2^) =.37 in organization; *F* (1, 68) = 26.69, *p* < .05, partial eta square (*η*^2^) =.28 in vocabulary; *F* (1, 68) =26.69, *p* < .05, partial eta square (*η*^2^) =.33 in language use. However, there was no significant difference found in mechanics when comparing students’ writing performance of the control and the experimental group. It is clear that the experimental group outperformed the control group after the intervention with large effect sizes in overall performance, content, organization, vocabulary and language use, indicating that the translation method proved to be effective in improving students’ writing performance.

### 4.3. Effect size

To examine the performance differences of students with different language proficiency, mean, SD of their two writing tests and effect sizes were estimated of the top and bottom 15 students of the experimental group. The results are shown in Table 4.

**Table 4:** Mean, SD, and effect sizes of writing performance of students with high and low language proficiency in the experimental group.

	First-test mean		SD		Second-test mean		SD		Effect size	
	Top	Bottom	Top	Bottom	Top	Bottom	Top	Bottom	Top	Bottom
Overall	68.13	63.75	7.36	8.88	74.67	73.27	4.32	2.89	1.08	1.44
Content	19.93	18.75	1.67	3.19	21.07	20.67	1.10	1.11	0.81	0.80
Organization	15.87	14.75	1.70	2.60	16.67	16.33	0.98	1.05	0.57	0.80
Vocabulary	14.27	13.58	1.624	1.73	16.40	16.07	1.18	0.70	1.50	1.89
Language use	14.87	13.75	2.50	2.53	16.93	16.60	1.22	0.91	1.05	1.50
Mechanism	3.20	2.92	0.86	0.79	3.60	3.60	0.51	0.51	0.57	1.02
Average	22.71	21.25	2.62	3.29	24.89	24.42	1.55	1.20	0.93	1.24

As shown in Table 4, the students with low and high proficiency all made progress in the overall performance and the five aspects of the essay with medium to large effect sizes. The bottom 15 students, i.e., the low proficiency group made more improvement than the high proficiency group. On the basis of the analysis results, the most improved areas for the low proficiency group include vocabulary (Cohen’s *d* = 1.89), language use (Cohen’s *d* = 1.50) and overall performance (Cohen’s *d* = 1.44) while the high proficiency group improved most in the areas of vocabulary (Cohen’s *d* = 1.50), overall performance (Cohen’s *d* = 1.08) and language use (Cohen’s *d* = 1.05) etc.

### 4.4. Analysis of the qualitative data

The qualitative data analysis was coded first and then frequency rates of the whole group and each of the high-, medium- and low-proficiency groups were calculated as shown in Table 5. The raw data were converted into ratio scores using a type/token analysis. That is, the frequency rate is the ratio of the number of occurrences of each coded category in relation to the total number of items of a type (Cohen and Upton, 2006, 2007). In other words, the higher the frequency rate is, the more positive the interviewer was toward the specific category.

**Table 5:** Frequency rate of the effect of translation methods as indicted in the qualitative data analysis.

The translation method improved my…	Overall frequency rate	High-proficiency group’s frequency rate	Medium-proficiency group’s frequency rate	Low-proficiency group’s frequency rate
Writing performance	0.89	0.67	1.00	1.00
Content	0.11	0.00	0.33	0.00
Organization	0.44	1.00	0.00	0.33
Language use	0.44	0.33	0.33	0.67
Vocabulary	0.89	1.67	0.33	0.67
Task-examining	0.11	0.33	0.00	0.00
Idea-generating	0.78	0.67	0.67	1.00
Idea-organizing	1.11	1.33	1.67	0.33
Text-generating	0.78	1.33	0.67	0.33
Confidence	1.00	1.00	1.33	0.67
Interest	0.89	1.00	0.67	1.00

Generally speaking, based on the frequency rate, the interviewed students viewed the translation method as a tool of improving their ability in forming thoughts into ideas and organizing them into words as well as generating new ideas. The translation method improved their writing performance, especially in terms of vocabulary use in essay writing. For example, the following is what one student of the high proficiency level mentioned.

“…if you encounter new words, you can search. Then there are a few hints in Chinese, and then your accumulation of new expressions will increase a little anyway, and then even if there are a lot of things that don’t, after you search this time, the next time, it will be regarded as accumulation.” (1a)

They believed that the translation method improved their writing performance in affective dimensions as well, especially in their confidence and interest in essay writing. For example, the following are what the students mentioned in their interview.

“Well, I have more interest, because it is easier to write with the translation method. When you write it out, there will definitely be a certain sense of accomplishment. When you have a certain sense of accomplishment, your confidence and interest in that writing will increase.” (1b)

“Yes, …, sometimes the biggest difficulty I face is that I may think it out in my mind, but I may not write it in English. If you practice more translation, your ability in this area will be improved.” (2a)

“Then it was helpful to boost my confidence, because I have ideas, and I only needs to do this step of translation, which will be easier.” (2b)

“… translation helps someone like me who has weak basic knowledge in English. …Because of these translation methods, I will master some new expressions or very good expressions, and I will try to use them the next time I write a composition. I have gained interest in writing in this way.” (3b)

“Yes, I become more interested in writing, because my English is relatively poor, and then if I use the translation method, it will make me have a sense of accomplishment.” (3c)

For the high-proficiency group, the translation method was viewed as an important means of improving their vocabulary use and organization in essay writing. In term of the writing process, the translation method improved their idea organization and text generation. They also believed that the translation method improved their confidence and interest in essay writing. For the medium-proficiency group, the translation method improved their idea-organizing and confidence in writing most. This group of students also thought that the translation method improved their idea-generation and text-generation as well as their interest in writing. For the low-proficiency group, they thought that the translation method enhanced their idea-generation and interest in writing most. In addition, their language use and vocabulary were improved after the translation method was used in the writing classes. Their confidence in writing was also increased with the use of the translation method.

## 5. Discussion

On the basis of the analysis results, this section discusses the findings in relation to the three research questions.

### 5.1. RQ1: Has the translation method improved Chinese college students’ writing performance in the linguistic dimension?

The result of ANCOVA indicated that the translation method was effective in improving the participants’ writing performance in general with a large effect size of 0.58 (*η*^2^). The finding is consistent with Lee’s (2018) in which the participants’ writing capacity was improved with the use of translation method. Specifically, students’ content, organization, vocabulary and language use all improved significantly compared with those in the control group. Among the four aspects, students improved their content the most with the greatest effect size of 0.75 (*η*^2^), followed by organization with 0.37 (*η*^2^), language use with 0.33 (*η*^2^) and vocabulary with.28 (*η*^2^). In other words, the translation methods seem to have improved students’ contents the most. This might be due to the fact that as L1 writers, these students can produce rich contents. However, their limited L2 proficiency constraints their output while writing English essays. The use of the translation method seems to have helped the students generate better contents in essay writing. Similarly, their organization was improved with better contents. For language use and vocabulary, it is understandable that students learned more new words and expressions while translating from L1 to L2.

The results of the qualitative data analysis supported the above-mentioned findings. For example, the three groups of students, i.e., high-, medium-, and low-proficiency groups all pointed out that the translation method improved their writing performance, especially in terms of vocabulary, language use and organization. With the help of the translation method, the low-proficiency students reported they could think of more ideas in essay writing. This clearly shows that L2 students with relatively low English proficiency were able to express their ideas in L2 in a better way with the help of the translation method. This finding is supported by precious research in this area (e.g., Wang and Wen, 2002; Kim, 2011; Lee, 2018). In addition, the medium- and high- proficiency students’ idea-organizing and text-generating skills were improved. This shows that the translation method has the potential to improve students’ writing process which will be very beneficial for the improvement of students’ writing performance in the long run. Similarly, researchers in the area of translanguaging pedagogies also reported that bilingual/multi-lingual resources enable students to activate their rich language resources and help generating rich texts (Gort, 2015; Gort and Sembiante, 2015; Jiménez et al., 2015; García and Kleifgen, 2019).

### 5.2. RQ2: How has the translation method affected the writing performance of the students with higher and lower language proficiency?

Generally speaking, all the top- and bottom-level participating students made improvement in their writing performance. As indicated in the effect sizes in Table 4, with the help of the translation method, the students with lower language proficiency made greater progress on average than those with high language proficiency. Their most improved area lies in vocabulary, followed by language use and overall performance. This finding shows that the translation method used in writing instruction was effective in enhancing the lower proficiency students’ language proficiency in terms of vocabulary and language use such as sentence structures and grammar, etc. The finding supports Kim (2011) in which the lower proficiency students improved their English writing through translation method and peer support. Similarly, Lee (2018) also found that the participants with lower ability improved their English proficiency after the translation-based EFL writing class.

As mentioned previously in discussion about RQ1, the translation method not only eradicates the barriers in writing, such as insufficient vocabulary, difficulty in generating contents etc., but also help improve the organization. For the lower proficiency students, they benefited from multiple aspects while using the translation method, including enriching content generating, improving language use, organization etc. The finding is congruent with the results of the qualitative data analysis as shown in Table 5, which clearly shows that the translation method improved low-proficiency students’ vocabulary and language use the most.

For the students with higher proficiency level, their weakness mainly lies in the vocabulary use as observed by the class instructor. Based on the analysis of Cohen’s *d*, the students with high language proficiency in this group improved their vocabulary the most. This is consistent with the results of the qualitative data analysis, as shown in the frequency rate analysis in Table 5. As indicated in the interview, the students with higher language proficiency mentioned that in the process of translation, they learned new words and expressions as they were given the chances to do search of some words and expressions on websites. The process made it easier for them to memorize the words and expressions. The other top students explained that with the translation process, they had the opportunities to compare different usage of one word or expression and then chose the most appropriate one. In a word, the translation method provided the students with the chances to focus more on the level of expressions, which can help them improve and refine their vocabulary and language use. In other words, the translation method may help improve their vocabulary use and as a result, their overall writing performance was improved.

### 5.3. RQ3: Did the participants perceive the translation method improved their writing in the affective dimension?

As shown in the frequency rate analysis in Table 5, all the high-, medium-, and low-proficiency groups of students reported that their confidence in writing English essays was improved. The highest frequency rate was reported by the medium-proficiency students followed by high- and low-proficiency students. All the three groups of students believed that their interest in essay writing was promoted.

As mentioned by the student, who is of high proficiency level in English, the process of writing in Chinese first and then translating into English gave them the chance to learn new vocabulary through self-study. In this way, their knowledge about vocabulary use has been accumulated, which demonstrates the potential power of translauguaging pedagogy (García and Kleifgen, 2019). In other words, the participating students felt that the translation method helped them overcome a major problem while writing English essays and made them feel easier to complete the essay writing. The whole process enhanced their confidence in writing English essays. In addition, the translation method seems to be effective in promoting the students’ interest in essay writing.

As indicated in students’ interview, no matter what proficiency level they are at in English, the participating students all mentioned that the whole writing process enhanced their interest in English essay writing. The results of the qualitative analysis also show that the use of translation method can have a very positive effect on students’ process of writing. For example, with the translation method, the low-proficiency students can generate ideas of writing easily. Their skills and strategies of idea-organizing and text-generating were also improved and enriched. This may explain why the translation method has improved their confidence and interest in essay writing. In summary, the findings show that the translation method has the potential to help students realize that as long as they can write in L1, it is likely that they can write in L2 through translation (Wang and Wen, 2002; Lee, 2018). In other words, EFL students’ first language can be a very important resource to be used in L2 classrooms (Atkinson, 1987).

According to Cenoz and Gorter (2021), pedagogical translanguaging “aims at improving language and content competences in school contexts by using resources from the learner’s whole linguistic repertoire.” (p.1) As a learner centered approach, pedagogical translanguaging supports the development of all the languages used by learners and can be a used as strategy for L2 learning and teaching. Findings from this study therefore provide an alternative method to improve L2 learners’ writing ability, interests and confidence etc.

## 6. Conclusion

This study investigates the effect of translation methods on Chinese EFL college students’ writing performance. Results shows that with the translation method, the participating students improved their essay writing significantly from the linguistic perspective. In addition, their confidence and interest in essay writing were improved. It seems that the students with low English proficiency made the greatest progress, especially in vocabulary and language use. The findings from this study provide very useful implications for effective writing instruction for EFL students, especially those with relatively low English proficiency. On this basis, a systematic translation-based writing instruction for EFL college students can be developed further (Lee, 2018).

As one of the pioneering studies in this area, the instruction method used in this study can be improved further in similar studies in future. For example, during the interview, the students suggested that the innovative teaching method should be combined with translation to avoid the dullness of the process of writing in L1 and translating it into L2. This is in line with Calis and Dikilitaş (2012) that “translation as a practice in EFL setting should be carefully designed and performed if effective results are expected” (p. 5080). Similarly, Carreres (2006) argued that the flexibility and innovation are very important to “build bridges between language teaching and translation pedagogy” (p. 18). Furthermore, with the development of pedagogical translanguaging (Cenoz and Gorter, 2021), L2 learners’ overall language resources are expected to be utilized more effectively, and the translation method in L2 writing instruction provides insights into the actual practice of pedagogical translanguaging in L2 classrooms.

In addition, there are also limitations to this study due to constraints in time and resources. For example, if time and conditions permit, questionnaire data could be collected from the participating students to have a deeper understanding of the effect of the translation method on their confidence and interest in essay writing. Thinking-aloud process could have also been used to see the students brainstorming for ideas and words for thoughts. It is hoped that future studies can have an improved design of the translation-based instruction method and implement it among a larger sample of students. That way, the findings of research will provide a more representative picture of the translation method in writing instruction and give useful implications for future similar instructional methods for EFL writing under the framework of pedagogical translanguaging.

## Data availability statement

The raw data supporting the conclusions of this article will be made available by the author, without undue reservation.

## Ethics statement

The studies involving human participants were reviewed and approved by Nanyang Technological University Ethical Review Committee. The participants provided their written informed consent to participate in this study.

## Author contributions

The author confirms being the sole contributor of this work and has approved it for publication.

## Conflict of interest

The author declares that the research was conducted in the absence of any commercial or financial relationships that could be construed as a potential conflict of interest.

## Publisher’s note

All claims expressed in this article are solely those of the author and do not necessarily represent those of their affiliated organizations, or those of the publisher, the editors and the reviewers. Any product that may be evaluated in this article, or claim that may be made by its manufacturer, is not guaranteed or endorsed by the publisher.

## Footnotes

^1^Second language (L2) is used as a superordinate term referring to any language learned after the first language (Ellis, 1994, 2003; Mokhtari et al., 2008).

## Appendix Interview questions

1. Do you think the translation method is useful to improve your writing performance? Why or why not?

你觉得翻译对你的写作表现有帮助吗?为什么?

2. At which stage of writing an essay do you think you used translation the most? task examining, idea generating, idea organising, text generating, text revising or process controlling? In what way dd the translation help you in each stage of your essay writing?

你觉得写作文的哪个阶段使用翻译最多,是审题、观点产生、观点组织、行文、修改还是整个写作过程的控制?对你写作文有什么帮助?

3. Do you think the translation method improved your confidence in writing English essays? Why or why not?

你觉得翻译对你的写作信心有帮助吗?为什么?

4. Do you think the translation method improved your interest in writing English essays? Why or why not?

你觉得翻译对你的写作兴趣有提升吗? 为什们?

5. Do you have any suggestions about how to use the translation in English essay writing?

你对使用翻译上英文作文课有什么建议?
